# Intra-individual comparison of coronary calcium scoring between photon counting detector- and energy integrating detector-CT: Effects on risk reclassification

**DOI:** 10.3389/fcvm.2022.1053398

**Published:** 2023-01-19

**Authors:** Elias V. Wolf, Moritz C. Halfmann, U. Joseph Schoepf, Emese Zsarnoczay, Nicola Fink, Joseph P. Griffith, Gilberto J. Aquino, Martin J. Willemink, Jim O’Doherty, Michaela M. Hell, Pal Suranyi, Ismael M. Kabakus, Dhiraj Baruah, Akos Varga-Szemes, Tilman Emrich

**Affiliations:** ^1^Department of Diagnostic and Interventional Radiology, University Medical Center of the Johannes Gutenberg-University, Mainz, Germany; ^2^Division of Cardiovascular Imaging, Department of Radiology and Radiological Science, Medical University of South Carolina, Charleston, SC, United States; ^3^German Centre for Cardiovascular Research, Partner Site Rhine-Main, Mainz, Germany; ^4^MTA-SE Cardiovascular Imaging Research Group, Medical Imaging Center, Semmelweis University, Budapest, Hungary; ^5^Department of Radiology, University Hospital Munich, LMU Munich, Munich, Germany; ^6^Department of Radiology, Stanford University School of Medicine, Stanford, CA, United States; ^7^Segmed, Inc., Palo Alto, CA, United States; ^8^Siemens Medical Solutions USA, Inc., Malvern, PA, United States; ^9^Department of Cardiology, University Medical Center of the Johannes Gutenberg-University, Mainz, Germany

**Keywords:** coronary artery calcium scoring (CACS), coronary artery disease, photon-counting detector computed tomography, risk stratification, energy-integrating detector computed tomography

## Abstract

**Purpose:**

To compare coronary artery calcium volume and score (CACS) between photon-counting detector (PCD) and conventional energy integrating detector (EID) computed tomography (CT) in a phantom and prospective patient study.

**Methods:**

A commercially available CACS phantom was scanned with a standard CACS protocol (120 kVp, slice thickness/increment 3/1.5 mm, and a quantitative Qr36 kernel), with filtered back projection on the EID-CT, and with monoenergetic reconstruction at 70 keV and quantum iterative reconstruction off on the PCD-CT. The same settings were used to prospectively acquire data in patients (*n* = 23, 65 ± 12.1 years), who underwent PCD- and EID-CT scans with a median of 5.5 (3.0–12.5) days between the two scans in the period from August 2021 to March 2022. CACS was quantified using a commercially available software solution. A regression formula was obtained from the aforementioned comparison and applied to simulate risk reclassification in a pre-existing cohort of 514 patients who underwent a cardiac EID-CT between January and December 2021.

**Results:**

Based on the phantom experiment, CACS_*PCD–CT*_ showed a more accurate measurement of the reference CAC volumes (overestimation of physical volumes: PCD-CT 66.1 ± 1.6% vs. EID-CT: 77.2 ± 0.5%). CACS_*EID–CT*_ and CACS_*PCD–CT*_ were strongly correlated, however, the latter measured significantly lower values in the phantom (CACS_*PCD–CT*_: 60.5 (30.2–170.3) vs CACS_*EID–CT*_ 74.7 (34.6–180.8), *p* = 0.0015, *r* = 0.99, mean bias –9.7, Limits of Agreement (LoA) –36.6/17.3) and in patients (non-significant) (CACS_*PCD–CT*_: 174.3 (11.1–872.7) vs CACS_*EID–CT*_ 218.2 (18.5–876.4), *p* = 0.10, *r* = 0.94, mean bias –41.1, LoA –315.3/232.5). The systematic lower measurements of Agatston score on PCD-CT system led to reclassification of 5.25% of our simulated patient cohort to a lower classification class.

**Conclusion:**

CACS_*PCD–CT*_ is feasible and correlates strongly with CACS_*EID–CT*_, however, leads to lower CACS values. PCD-CT may provide results that are more accurate for CACS than EID-CT.

## 1. Introduction

Coronary artery calcium scoring (CACS) is an established method to assess the presence and extent of coronary artery calcifications (1). CACS is an integral part of several guidelines for risk assessment of coronary artery disease (CAD) in asymptomatic and symptomatic individuals (2, 3).

Recently, a first-generation dual-source photon-counting detector (PCD) computed tomography (CT) with electrocardiogram (ECG) gating capability became clinically available. Compared to conventional energy-integrating detector (EID) CT, PCD-CT directly converts x-ray photons into an electrical signal without a conversion into light. This difference leads to potential advantages of PCD-CT in relation to spatial resolution and image noise. As the PCD-CT detector “counts” each individual photon in relation to its energy, it is thus capable of providing spectral image information for every acquired scan (4). CACS has been investigated in phantom studies using PCD-CT against EID-CT, combining specific settings of different slice thicknesses, quantum iterative reconstruction (QIR) levels, and virtual monoenergetic image (VMI) reconstruction levels (5, 6). However, to the best of our knowledge, no data exists comparing intra-individual CACS between EID- and PCD-CT in patients according to the Agatston standard. In addition, the effect of PCD-CT-based CACS on reclassification rates for risk prediction is unknown.

Hence, the aim of this study was to compare CACS between PCD-CT and conventional EID-CT in a commercially available phantom with known plaque volumes and calcium densities, as well as in a prospective patient cohort. A further aim was to evaluate the possible effects of PCD-CT-based CACS on reclassification of cardiovascular risk.

## 2. Materials and methods

### 2.1. Phantom study

A commercially available chest phantom with a cardiac calcification insert (Thorax & Cardiac Calcification Phantom, QRM, Moehrendorf, Germany) was used as the ground truth for the *in vitro* study. The phantom contains nine different cylinders with predefined plaque diameter (small: 1 mm; medium: 3 mm; and large: 5 mm), each with three different densities (low-density: 200 mg/cm^3^; medium-density: 400 mg/cm^3^, and high-density: 800 mg/cm^3^ calcium hydroxyapatite). The thoracic phantom simulates a small-sized patient (anterior posterior and lateral diameter: 200 and 300 mm) and was used without extension rings.

### 2.2. Phantom data acquisition

Phantom measurements were performed on both a conventional EID-CT (SOMATOM Force, Siemens Healthineers, Forchheim, Germany) and a PCD-CT system (NAEOTOM Alpha, Siemens Healthineers). The PCD-CT contains two photon-counting cadmium telluride (CdTe) detectors with 144 × 0.4 mm collimation on each detector, allowing spectral CT data acquisition at a maximum temporal resolution of 66 ms. The EID-CT is equipped with a detector collimation of 192 × 0.6 mm. For both systems, tube voltage was set to 120 kVp and volumetric CT Dose Index (CTDI_*vol*_) was matched between the CT systems at 1.5 mGy. ECG signal was simulated at 60 beats per minute and the examination was triggered at the diastolic phase (75% of the cardiac cycle). To account for variability of the different scans, phantom measurements were repeated five times with a 2 mm shift and a 2° rotation (7).

A standard CACS CT protocol in sequential mode with a slice thickness of 3 mm, increment of 1.5 mm and a quantitative kernel (Qr36) was used on both systems. Images were reconstructed using filtered back projection on the EID-CT. For the PCD-CT, CACS scans were acquired according to a protocol suggested by the manufacturer with a monoenergetic reconstruction at 70 keV and the lowest available level of quantum iterative reconstruction (QIR off). Detailed acquisition and reconstruction parameters are listed in Table 1.

**TABLE 1:** CT acquisition and reconstruction parameter.

Modality	EID-CT	PCD-CT
Tube potential (kVp)	120	120
Monoenergetic level (keV)	n/a	70
Reconstruction technique	Filtered back-projection	QIR off
Reconstruction kernel	Qr36f	Qr36f
Slice thickness/Increment (mm)	3/1.5	3/1.5
Field of view (mm)	200	200
Matrix size	512 × 512	512 × 512

QIR, quantum iterative reconstruction.

### 2.3. Patient study

The protocol of this prospective, HIPAA-compliant, study was approved by the local Institutional Review Board and written informed consent was obtained from all subjects. All patients underwent standard of care imaging on an EID-CT system, and an additional research CT scan on a PCD-CT system between August 2021 and March 2022. The following inclusion criteria were applied: (1) clinical indication for cardiac imaging, and (2) >18 years of age. Exclusion criteria was: unable to be consented.

### 2.4. Patient data acquisition

CACS scans were acquired according to the phantom experiment using the same two CT systems. CTDI_*vol*_ were adjusted to be as similar as possible between PCD-CT and EID-CT, and based on the automatic dose selection of the patient at the EID-CT (CareDose4D, Siemens Healthineers). Reconstruction parameters were identical to the phantom experiment.

### 2.5. Calcium scoring

**FIGURE 1:**
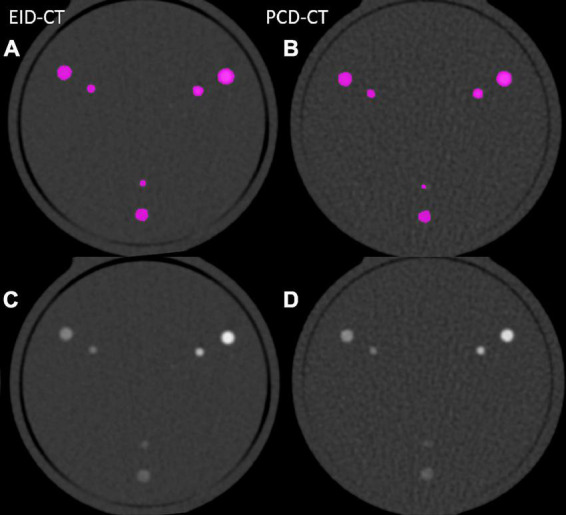
Image examples from phantom scanned in the EID-CT **(A,C)** and PCD-CT **(B,D)** with and without color-overlay for CACS.

**FIGURE 2:**
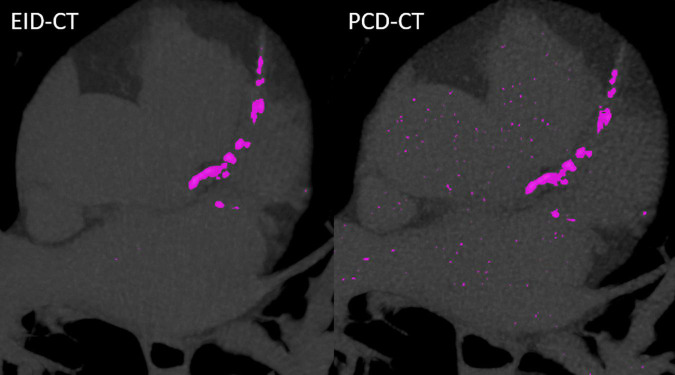
Case example from a patient scanned in the EID-CT (CACS_*EID–CT*_: 964.1) and PCD-CT (CACS_*PCD–CT*_: 949.9).

*In vitro* and *in vivo* CACS was performed using a commercially available software solution (CT CaScoring, syngo.via Version VB60, Siemens Healthineers) by a single reader with 2 years of experience in cardiovascular radiology, under the supervision of a board-certified cardiovascular radiologist with 12 years of experience. The reader was blinded to the origin of the CACS image. The software solution semi-automatically quantifies coronary artery calcium (CAC) volumes and CACS according to Agatston’s method. The algorithm uses a threshold of 130 HU at 120 kVp and a minimum of a 0.5 mm^2^ connected area to detect calcium-containing voxels (8). Segments with stents, or affected by artifacts related to pacemakers, or other metallic objects, were manually excluded. Representative phantom and patient examples are given in Figures 1, 2. For risk assessment, the Rumberger classification was used to stratify CACS into the following classes: 0, 1–10, 11–100, 101–400, and >400 (9).

### 2.6. Reclassification simulation

A regression model was generated based on the CACS comparison derived from the patient and phantom data to facilitate a virtual recalculation and reclassification in a simulation study as described previously (10). PCD-CT and EID-CT were compared, and the EID-CT was considered as the clinical reference standard. Therefore, a zero value in the EID-CT also causes a non-detectable value in the PCD-CT and there are no negative Agatston scores. Thus, a linear trend line with a y-intercept of zero was fitted to describe inter-scanner CACS differences. A retrospective patient cohort, who had previously undergone cardiac imaging on the same EID-CT between January and December 2021, was used to investigate the effect on the reclassification rate in a larger cohort. Inclusion criteria were an Agatston score above zero. Hence, a total of 514 out of 1,301 screened patients were included in the simulation study.

### 2.7. Statistical analysis

Statistical analysis was performed using dedicated software (SPSS Statistics for Windows, Version 21.0, IBM Corp Armonk, NY, and MedCalc for Windows, version 15.0, MedCalc Software, Ostend, Belgium). Mean ± standard deviations (SD) were used for normally distributed and median with an interquartile range for non-normal distributed data. Categorical variables are reported with frequencies and proportions. The difference between the CT measurements were compared with the Wilcoxon rank sum tests. A *p*-value ≤ 0.05 was considered significant. Spearman correlation coefficient (*r*) was used to assess the correlation between CACS by PCD-CT and EID-CT. Through Bland-Altman analyses, the mean bias, and the upper and lower limits of agreement (LoA) between the two CT techniques were assessed. Intraclass correlations coefficients (ICC) were used to measure the agreement between the two different CT scanners with the following interpretation: 0.0–0.3, lack of agreement; 0.31–0.5, weak agreement; 0.51–0.7, moderate agreement; 0.71–0.9, strong agreement; and 0.91–1.00, very strong agreement (11).

## 3. Results

### 3.1. Phantom study

**FIGURE 3:**
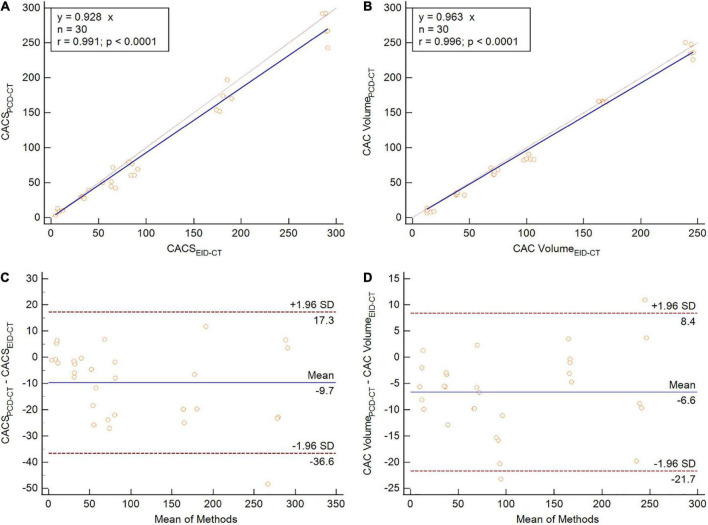
Phantom study comparing CACS **(A,C)** and CAC volume **(B,D)** between PCD-CT and EID-CT in scatter- **(A,B)** and Bland-Altman plots **(C,D)**. SD, standard deviation; *r*, Spearmen correlation.

**FIGURE 4:**
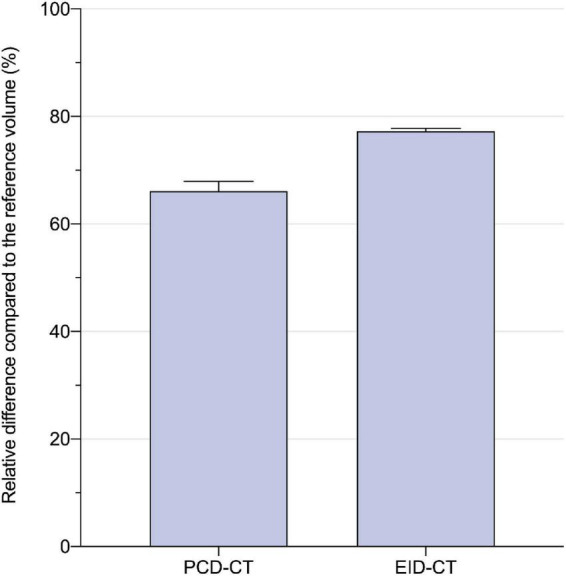
Relative difference to the physical calcium insert volume (in percent) for PCD- and EID-CT: 66.1 ± 1.6 vs. 77.2 ± 0.5%.

CACS_*PCD–CT*_ showed a strong correlation to CACS_*EID–CT*_, however, CACS_*PCD–CT*_ measured significantly lower values compared to CACS_*EID–CT*_ (CACS_*PCD–CT*_: 60.5 (30.2–170.3) vs CACS_*EID–CT*_ 74.7 (34.6–180.8), *p* = 0.0015, *r* = 0.99, ICC = 0.99, mean bias –9.7, LoA –36.6/17.3) (Figure 3). Compared to the physical volumes of the calcium inserts, PCD-CT had a lower overestimation to the reference value (overestimation of PCD-CT: 66.1 ± 1.6% vs EID-CT: 77.2 ± 0.5%) (Figure 4).

### 3.2. Patient study

The study population consisted of 23 patients (65 ± 12.1 years) including 16 men and 7 women who underwent CACS scans on both CT scanners. The EID-CT scan was followed by the PCD-CT scan after a median of 5.5 (3.0–12.5) days. Radiation dose (CTDIvol) was lower in PCD-CT imaging compared to the EID-CT (CTDI_*vol*_ 2.4 (1.5–3.1) mGy and 2.9 (1.7–3.1) mGy, *p* = 0.0001). Details of the study population are given in Table 2.

**TABLE 2:** Patient characteristics.

	EID-CT	PCD-CT
*N*	23	
Female (%)	7 (30.4)	
Age (years)	65 ± 12.1	
BMI (kg/m^2^)	28.6 (26.7–31.3)	
Difference between both scans (days)	5.5 (3.0–12.5)	
Heart rate (bpm)	66.6 ± 14.5^#^	62.0 (55.0–73.0)^#^
CTDI_*vol*_ (mGy)	2.9 (1.7–3.1)*	2.4 (1.5–3.1)*
DLP (mGy*cm)	45.0 (33.3–61.0)*	33.4 (24.4–48.5)*

Values are mean ± standard deviation, median (interquartile range), absolute and relative frequencies.

BMI, body mass index; CTDI, computed tomography dose index; DLP, dose length product.

Comparison between EID- and PCD-CT: ^#^*p* = 0.82, *p < 0.001.

**FIGURE 5:**
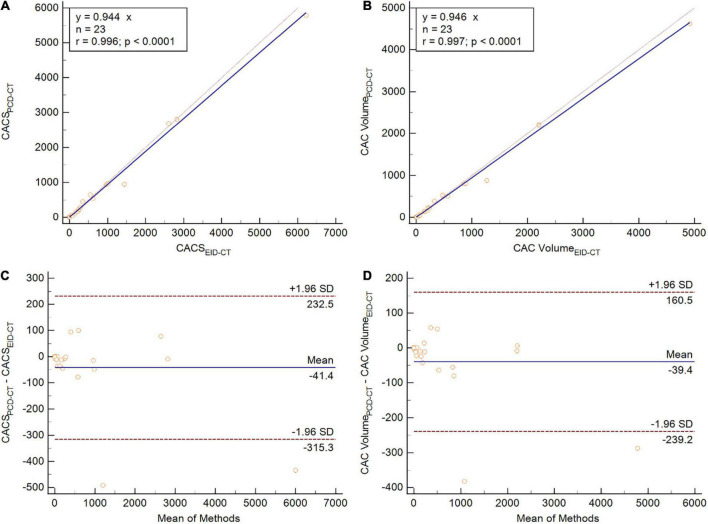
Patient study comparing CACS **(A,C)** and CAC volume **(B,D)** between PCD-CT and EID-CT in scatter- **(A,B)** and Bland-Altman plots **(C,D)**. SD, standard deviation; *r*, Spearmen correlation.

Similar to the phantom studies, the *in vivo* comparison yielded strong correlation between PCD-CT and EID-CT for CACS (*r* = 0.94, ICC = 0.99). When comparing median CACS, PCD-CT showed lower CACS then EID-CT, but did not meet statistical significance (CACS_*PCD–CT*_: 174.3 (11.1–872.7) vs CACS_*EID–CT*_ 218.2 (18.5–876.4), *p* = 0.10, mean bias –41.1, LoA –315.3/232.5) (Figure 5). Global CACS volume was significantly lower for PCD-CT (*p* = 0.04). Detailed comparisons for the entire heart and per vessel are shown in Table 3.

**TABLE 3:** Comparison of total and per-vessel values between EID-CT and PCD-CT in patients.

	EID-CT	PCD-CT	*p*	*r*	ICC	Bias	LoA
Total CACS	218.2 (18.5–876.4)	174.3 (11.1–872.7)	0.10	0.99	0.99	–41.4	–315.3/232.6
Total CAC volume	206.8 (15.9–782.6)	165.0 (9.8–731.6)	0.04	0.99	0.99	–37.6	–233.6/158.3
LM CACS	0.0 (0–21)	0.0 (0–26.9)	0.38	0.98	0.94	–5.6	–45.7/34.6
LM CAC volume	0.0 (0–19.1)	0.0 (0–19.1)	0.46	0.99	0.97	–3.0	–26.2/20.1
LAD CACS	91.0 (0.6–266.9)	96.3 (0.7–273.5)	0.22	0.99	0.99	16.1	–80.3/112.4
LAD CAC volume	78.0 (1.5–205.7)	83.1 (1.4–218.6)	0.35	0.99	0.99	8.2	–44.3/60.7
LCX CACS	53.1 (0–297.5)	16.7 (0–292.4)	0.11	0.97	0.95	–49.5	–356.1/257.0
LCX CAC volume	9.5 (0–47.3)	7.9 (0–45.4)	0.41	0.99	0.99	–0.2	–37.3/37.0
RCA CACS	53.1 (0–297.4)	16.7 (0–292.4)	0.30	0.97	0.95	–49.5	–356.1/257.0
RCA CAC volume	64.6 (0–291.7)	28.3 (0–277.2)	0.08	0.97	0.95	–41.2	–283.2/200.7

Values are median (interquartile range).

LM, left main, LAD, left anterior descending; LCX, left circumflex; RCA, right coronary artery; CACS, coronary artery calcium scoring; CAC, coronary artery calcium; ICC, intraclass correlation; LoA, limits of agreement.

### 3.3. Reclassification simulation

The regression equation from *in vitro* and *in vivo* CACS comparison (CACS_*PCD–CT*_ vs. CACS_*EID–CT*_; *y* = 0.936*x*) was used to calculate a new risk classification for PCD-CT. From 1,301 patients, 787 patients had an Agatston score of 0 and 514 patients an Agatston score above zero. From these 514 patients, 5.25% (27 patients) were reclassified in a new, lower CACS risk score classification. Details are provided in Table 4.

**TABLE 4:** Reclassification study for simulated PCD-CT examinations.

	Original risk classification	New risk classification	Difference (%)
**Agatston score category**			
0	787	0	
1–10	62	5	8.06
11–100	132	5	3.79
101–400	144	10	6.94
>400	176	7	3.98
Total (>0)	514	27	5.25
Total	1,301		

Total number of patients (n).

## 4. Discussion

In this study, we investigated the intra-individual difference in CACS between PCD- and EID-CT in a phantom and a patient cohort. The major findings are: First, PCD-CT measures lower CACS values compared to EID-CT in an intra-individual *in vitro* and *in vivo* comparison. Second, *in vitro* experiments demonstrated that PCD-CT resulted in more accurate quantification of calcium volume. Third, the reclassification rate for risk prediction was 5.25% using a simulation study of 514 patients who underwent a CACS EID-CT examination.

Various histopathological studies have shown a relevant correlation between coronary calcifications and arteriosclerotic disease (12, 13). CACS has a high negative predictive value, which carries along a low risk for cardiovascular diseases (14, 15). Hence, CACS is a useful diagnostic tool to stratify patients into low and intermediate/high-risk groups. Furthermore, CACS can be used as a prognostic tool predicting cardiovascular events and death (16, 17).

Our results from the phantom study suggest that PCD-CT measures coronary artery calcium (CAC) more accurate than EID-CT. Overestimation of CAC volumes compared to the ground-truth is a widely known phenomenon. For example, van der Werf et al. measured higher CACS values in a phantom using an EID-CT compared to a prototype PCD-CT (6). An overestimation up to 150% of the CAC volume was measured for the large-volume and high-density calcium insert. This overestimation is caused by blooming artifacts that increase the measurable size of plaques (18). Intrinsically, measurements with the PCD-CT system seem to benefit from a reduction of blooming artifacts, even when reconstructed identically to the Agatston standard method derived from EID-CTs. Similarly to phantom experiments that demonstrate lower CACS by PCD-CT compared to EID-CT, our *in vivo* study also produced lower CACS values, despite not being statistically significant, (*p* = 0.10), which may be caused by our small patient sample. These results indicate that PCD-CT derived CACS is more accurate than EID-CT derived CACS with reference to the physical calcification volume.

Other investigations have compared CACS derived by PCD-CT using different settings: Eberhard et al. found that the settings of 70 keV and QIR off; 65 keV and QIR 3/QIR4; Polychromatic images (T3D) at 120 kV and QIR 4 have a <1% deviation from the reference CACS value (5). Additionally, other scan modes such as Sn100 kV with 70 keV and QIR 1 or at 90 kV with 65 keV and QIR 4 could be used to obtain CACS with similar accuracy to the EID-CT and lower radiation dose levels. (19, 20). Furthermore, CACS has also been successfully derived from CCTA datasets by using a novel virtual non-iodine reconstruction (PureCalcium) with high agreement to the true non-contrast acquisition (21).

Despite its clinical and prognostic relevance, CACS is prone to certain limitations (22). One of those is the limited repeatability of CACS on different CT systems, as demonstrated by Willemink et al. in cadaveric hearts (10). Reclassification rate may reach up to 6.5% of cases, depending on the vendor of the CT system. In addition, a certain inter-scan variability also exists when using a CT system for repeated measurements (23). This can be caused by several factors including partial volume effects, different breath-hold depth, and heart rate variability (24). Budoff et al. demonstrated a variability of 11.8% of the Agatston score in patients with end-stage renal disease, especially those with an Agatston score below 30 (inter-scan variability up to 61.3%).

The results of this study demonstrated a ∼5% lower CACS derived by PCD-CT compared to EID-CT. This inter-system deviation is in the range of prior reported inter-scan variabilities and indicates that PCD-CT can be used for CACS in clinical routine (23). However, our study investigated patients who underwent CACS on the EID-CT and simulated their virtual CACS risk class that would have been derived by the PCD-CT. According to our *in vitro* and *in vivo* evaluations, approximately 5% of patients would have been reclassified to a lower risk category with potential effects on clinical management such as initiation of optimal medical therapy. The new classification mostly affected patients who were classified immediately above the next category’s cut off. One should thus be cautious on the risk of potential reclassification when measuring CACS on PCD-CT images. To compare CACS from PCD- to a dual-source EID-CT (of the same vendor), CACS_*PCD–CT*_ may be multiplied by the factor of 1.056. Effects on prognostic and therapeutic implications and a potential change in risk stratification classes, however, have to be studied in future studies in larger cohorts.

Several attempts have been made to improve the conventional CACS protocol since its introduction. Recently, van Praagh et al. evaluated the optimization of several parameters in CACS for EID-CT systems from four different vendors compared to the standard CACS protocol (24). They reduced tube voltage to 100 kVp, radiation dose to 75%, slice thickness to 1 or 1.25 mm and applied a higher iterative reconstruction level resulting in a 36 and 34% lower intra- and inter-scan variability and improved detection of small and low-density calcifications. However, these protocol modifications have not been applied to PCD-CT derived CACS yet.

There are some limitations to our study that need to be considered: First, the distribution of calcifications was imbalanced in the patient cohort. There were only a limited number of patients with severe calcification. Second, our patient cohort was limited to 23 patients and a larger study group would be desirable to improve the statistics of the model extended to the reclassification rates. Clinical implications such as new reclassification classes for PCD-CT based CACS have to be investigated in further studies. Third, PCD-CT scans were acquired at slightly lower radiation dose levels, thereby theoretically making them more prone to software detection errors as CACS depends on acquisition and reconstruction settings and the corresponding image noise levels. Low dose scans with high image noise may result in higher Agatston scores, because noise pixels exceeding the 130 HU threshold may be erroneously counted as calcifications. In addition, the in this study used lowest setting of iterative reconstruction (QIR off) may not the optimal reconstruction setting for PCD-CT based CACS. However, CACS_*PCD–CT*_ systematically demonstrated lower CACS values in this study. Fourth, a ground truth for CACS was available only for phantom measurements. Differences and reclassification of *in vivo* data can therefore be interpreted only in relation to the phantom experiments, and we are unable to demonstrate which *in vivo* CACS measurement is more accurate. Fifth, differences in heart rate, radiation dose and other acquisition parameters including breath-hold and positioning could have affected the results. However, the analysis demonstrated systemic relationships that are unlikely caused by individual factors. Finally, we considered just one available phantom size, therefore, our results may not be applicable to different body types.

In conclusion, PCD-CT measures lower CACS values in both phantom and patient studies, resulting in reclassification of approximately 5% of individuals into lower risk groups. Compared to EID-CT, PCD-CT may be more accurate for physical quantification of calcifications. Further studies have to be conducted to evaluate optimized CACS PCD-CT imaging protocols and their potential effect on risk stratification and medical treatment.

## Data availability statement

The raw data supporting the conclusions of this article will be made available by the authors, without undue reservation.

## Ethics statement

The studies involving human participants were reviewed and approved by Institutional Review Board, Medical University of South Carolina, Charleston, SC, United States. The patients/participants provided their written informed consent to participate in this study.

## Author contributions

EVW, MCH, and TE designed the study, interpreted the study data, and drafted the manuscript. PS, IMK, GJA, and DB acquired the data and substantially revised the manuscript. EZ, NF, JPG, and MMH performed the data analysis, supported the statistical analysis, and substantially revised the manuscript. JO’D and MJW advised the data reconstruction, supervised the data analysis, and edited/revised the manuscript. AV-S and UJS supervised the study conception and data interpretation and substantially edited the manuscript. All authors read and approved the final manuscript.

## References

[1] Divakaran S Cheezum M Hulten E Bittencourt M Silverman M Nasir K Use of cardiac CT and calcium scoring for detecting coronary plaque: implications on prognosis and patient management. *Br J Radiol.* (2015) 88:20140594. 10.1259/bjr.20140594 25494818 PMC4614250

[2] Greenland P Alpert J Beller G Benjamin E Budoff M Fayad Z 2010 ACCF/AHA guideline for assessment of cardiovascular risk in asymptomatic adults: executive summary: a report of the American college of cardiology foundation/American heart association task force on practice guidelines. *Circulation.* (2010) 122:2748–64. 10.1161/CIR.0b013e3182051bab 21098427

[3] Goff D Jr Lloyd-Jones D Bennett G Coady S D’Agostino R Gibbons R 2013 ACC/AHA guideline on the assessment of cardiovascular risk: a report of the American college of cardiology/American heart association task force on practice guidelines. *Circulation.* (2014) 129(25 Suppl. 2):S49–73. 10.1161/01.cir.0000437741.48606.98 24222018

[4] Willemink M Persson M Pourmorteza A Pelc N Fleischmann D. Photon-counting CT: technical principles and clinical prospects. *Radiology.* (2018) 289:293–312. 10.1148/radiol.2018172656 30179101

[5] Eberhard M Mergen V Higashigaito K Allmendinger T Manka R Flohr T Coronary calcium scoring with first generation dual-source photon-counting CT-first evidence from phantom and in-vivo scans. *Diagnostics.* (2021) 11:1703. 10.3390/diagnostics11091708 34574049 PMC8466604

[6] van der Werf N Si-Mohamed S Rodesch P van Hamersvelt R Greuter M Boccalini S Coronary calcium scoring potential of large field-of-view spectral photon-counting CT: a phantom study. *Eur Radiol.* (2022) 32:152–62. 10.1007/s00330-021-08152-w 34255159 PMC8660747

[7] Groen J Greuter M Vliegenthart R Suess C Schmidt B Zijlstra F Calcium scoring using 64-slice MDCT, dual source CT and EBT: a comparative phantom study. *Int J Cardiovasc Imaging.* (2008) 24:547–56. 10.1007/s10554-007-9282-0 18038190 PMC2373860

[8] Agatston A Janowitz W Hildner F Zusmer N Viamonte M Jr Detrano R. Quantification of coronary artery calcium using ultrafast computed tomography. *J Am Coll Cardiol.* (1990) 15:827–32. 10.1016/0735-1097(90)90282-T 2407762

[9] Obisesan O Osei A Uddin S Dzaye O Blaha M. An update on coronary artery calcium interpretation at chest and cardiac CT. *Radiol Cardiothorac Imaging.* (2021) 3:e200484. 10.1148/ryct.2021200484 33778659 PMC7977732

[10] Willemink M Vliegenthart R Takx R Leiner T Budde R Bleys R Coronary artery calcification scoring with state-of-the-art CT scanners from different vendors has substantial effect on risk classification. *Radiology.* (2014) 273:695–702. 10.1148/radiol.14140066 25153157

[11] Koo T Li MYA. Guideline of selecting and reporting intraclass correlation coefficients for reliability research. *J Chiropr Med.* (2016) 15:155–63. 10.1016/j.jcm.2016.02.012 27330520 PMC4913118

[12] Oliver M Samuel E Morley P Young G Kapur P. Detection of coronary-artery calcification during life. *Lancet.* (1964) 1:891–5. 10.1016/S0140-6736(64)91625-3 14124078

[13] Sangiorgi G Rumberger J Severson A Edwards W Gregoire J Fitzpatrick L Arterial calcification and not lumen stenosis is highly correlated with atherosclerotic plaque burden in humans: a histologic study of 723 coronary artery segments using nondecalcifying methodology. *J Am Coll Cardiol.* (1998) 31:126–33. 10.1016/S0735-1097(97)00443-9 9426030

[14] Budoff M Diamond G Raggi P Arad Y Guerci A Callister T Continuous probabilistic prediction of angiographically significant coronary artery disease using electron beam tomography. *Circulation.* (2002) 105:1791–6. 11956121 10.1161/01.cir.0000014483.43921.8c

[15] Blaha M Cainzos-Achirica M Greenland P McEvoy J Blankstein R Budoff M Role of coronary artery calcium score of zero and other negative risk markers for cardiovascular disease: the multi-ethnic study of atherosclerosis (MESA). *Circulation.* (2016) 133:849–58. 10.1161/CIRCULATIONAHA.115.018524 26801055 PMC4775391

[16] Carr J Jacobs D Jr Terry J Shay C Sidney S Liu K Association of coronary artery calcium in adults aged 32 to 46 years with incident coronary heart disease and death. *JAMA Cardiol.* (2017) 2:391–9. 10.1001/jamacardio.2016.5493 28196265 PMC5397328

[17] Detrano R Guerci A Carr J Bild D Burke G Folsom A Coronary calcium as a predictor of coronary events in four racial or ethnic groups. *N Engl J Med.* (2008) 358:1336–45. 10.1056/NEJMoa072100 18367736

[18] Hoffmann U Ferencik M Cury R Pena A. Coronary CT angiography. *J Nucl Med.* (2006) 47:797–806. 16644750

[19] Mergen V Higashigaito K Allmendinger T Manka R Euler A Alkadhi H Tube voltage-independent coronary calcium scoring on a first-generation dual-source photon-counting CT-a proof-of-principle phantom study. *Int J Cardiovasc Imaging.* (2022) 38:905–12. 10.1007/s10554-021-02466-y 34780012

[20] van der Werf N van Gent M Booij R Bos D van der Lugt A Budde R Dose reduction in coronary artery calcium scoring using mono-energetic images from reduced tube voltage dual-source photon-counting CT data: a dynamic phantom study. *Diagnostics.* (2021) 11:2192. 10.3390/diagnostics11122192 34943428 PMC8699960

[21] Emrich T Aquino G Schoepf U Braun F Risch F Bette S Coronary computed tomography angiography-based calcium scoring: in vitro and in vivo validation of a novel virtual noniodine reconstruction algorithm on a clinical, first-generation dual-source photon counting-detector system. *Invest Radiol.* (2022) 57:536–43. 10.1097/RLI.0000000000000868 35318969

[22] Willemink M van der Werf N Nieman K Greuter M Koweek L Fleischmann D. Coronary artery calcium: a technical argument for a new scoring method. *J Cardiovasc Comput Tomogr.* (2019) 13:347–52. 10.1016/j.jcct.2018.10.014 30366859

[23] Budoff M Kessler P Gao Y Qunibi W Moustafa M Mao S. The interscan variation of CT coronary artery calcification score: analysis of the calcium acetate renagel comparison (CARE)-2 study. *Acad Radiol.* (2008) 15:58–61. 10.1016/j.acra.2007.08.011 18078907

[24] van Praagh G Wang J van der Werf N Greuter M Mastrodicasa D Nieman K Coronary artery calcium scoring: toward a new standard. *Invest Radiol.* (2022) 57:13–22. 10.1097/RLI.0000000000000808 34261083 PMC10072789

